# Retraction Note: The circular RNA 001971/miR-29c-3p axis modulates colorectal cancer growth, metastasis, and angiogenesis through VEGFA

**DOI:** 10.1186/s13046-022-02342-0

**Published:** 2022-03-29

**Authors:** Chen Chen, Zhiguo Huang, Xiaoye Mo, Yanmin Song, Xiangmin Li, Xiaogang Li, Mu Zhang

**Affiliations:** 1 Department of Pediatrics, Xiangya Hospital, Central South University, Changsha, Hunan People’s Republic of China; 2 Department of Emergency, Xiangya Hospital, Central South University, Changsha, Hunan People’s Republic of China

**Retraction Note: J Exp Clin Cancer Res 39, 91 (2020)**

**https://doi.org/10.1186/s13046-020-01594-y**

The Editor-in-Chief has retracted this article. Concerns were raised regarding the ethics approval as described in this article. The authors have been unable to provide documentation that confirms that the research as described in the article was approved by an IRB prior to the start of the study.

Authors have not responded to any correspondence from the publisher about this retraction.

